# Homology modeling and ligand docking of Mitogen-activated protein kinase-activated protein kinase 5 (MK5)

**DOI:** 10.1186/1742-4682-10-56

**Published:** 2013-09-14

**Authors:** Inger Lindin, Yimingjiang Wuxiuer, Irina Kufareva, Ruben Abagyan, Ugo Moens, Ingebrigt Sylte, Aina Westrheim Ravna

**Affiliations:** 1 Medical Pharmacology and Toxicology, Department of Medical Biology, Faculty of Health Sciences, University of Tromsø, Tromsø No-9037, Norway; 2 UCSD Skagg´s School of Pharmacy & Pharmaceutical Sciences, 9500 Gilman Drive, La Jolla CA 92093, USA; 3 Research Group for Molecular Inflammation, Department of Medical Biology, Faculty of Health Sciences, University of Tromsø, Tromsø No-9037, Norway

**Keywords:** MAPKAPK5, PRAK, ICM program package, Homology modeling, Docking, Molecular dynamics

## Abstract

**Background:**

Mitogen-activated protein kinase-activated protein kinase 5 (MK5) is involved in one of the major signaling pathways in cells, the mitogen-activated protein kinase pathway. MK5 was discovered in 1998 by the groups of Houng Ni and Ligou New, and was found to be highly conserved throughout the vertebrates. Studies, both in vivo and in vitro, have shown that it is implicated in tumor suppression as well as tumor promotion, embryogenesis, anxiety, locomotion, cell motility and cell cycle regulation.

**Methods:**

In order to obtain a molecular model of MK5 that can be used as a working tool for development of chemical probes, three MK5 models were constructed and refined based on three different known crystal structures of the closely related MKs; MK2 [PDB: 2OZA and PDB: 3M2W] and MK3 [PDB: 3FHR]. The main purpose of the present MK5 molecular modeling study was to identify the best suited template for making a MK5 model. The ability of the generated models to effectively discriminate between known inhibitors and decoys was analyzed using receiver operating characteristic (ROC) curves.

**Results:**

According to the ROC curve analyzes, the refined model based on 3FHR was most effective in discrimination between known inhibitors and decoys.

**Conclusions:**

The 3FHR-based MK5 model may serve as a working tool for development of chemical probes using computer aided drug design. The biological function of MK5 still remains elusive, but its role as a possible drug target may be elucidated in the near future.

## Background

Mitogen-activated protein kinases (MAPKs) constitute a major signaling pathway in cells, and are involved in processes controlling gene expression, cell division, cell survival, apoptosis, metabolism, differentiation and motility [1]. The conventional mammalian pathway consists of a cascade of three serine/threonine kinases referred to as MAPK kinase kinase, MAPK kinase and MAPK. The MAPKs are divided into four different subfamilies; the extracellular signal-regulated kinases 1/2 (ERK1/2), the c-JUN N-terminal kinases 1–3 (JNK1-3) or stress-activated protein kinases (SAPKα, β and γ), the p38 MAPKs (p38 α, β, γ and δ), and the big MAPKs (BMK1/ERK5). The atypical MAPK pathway is not organized in the normal three tier cascade, and includes ERK3/4, ERK7/8 and Nemo-like kinase (NLK) [1]. Both the conventional and the unconventional pathway can phosphorylate protein substrates and other protein kinases called mitogen-activated protein kinase-activated protein kinases (MAPKAPK). The MAPKAPK family compromises 11 Ser/Thr kinases and according to sequence similarities they can be subdivided into four groups: ribosomal S6 kinases (RSK1-4), mitogen and stress activated kinases (MSK1 and 2), MAPK-interacting kinases (MNK1 and 2) and MKs. The latter includes the members MK2, MK3, and MK5 [2].

The present study focuses on mitogen-activated protein kinase-activated protein kinase 5 (MK5). No analog to the *mapkapk-5* gene seems to be present in invertebrates or plants, but the vertebrate MK5 protein is a highly conserved protein kinase within vertebrates sharing 87 to 99% amino acid identity with the human MK5. Its molecular weight is 54,220 Da, and it is believed to be activated by both the conventional and unconventional MAPK pathways [3]. MK5 was originally discovered in 1998 by the research group of Houng Ni, as a novel murine kinase that could be phosphorylated and activated by ERK and p38 but not by Jun N-terminal kinase (JNKs) in vitro [4]. Later the research group of Ligou New also described a protein kinase activated downstream of p38 MAP kinase and called it PRAK. This was the human analog of MK5 [5].

MK5/PRAK shares 42% overall amino acid identity with MK2 (with a 48% similarity in the catalytic domain) and for MK5/MK3 the overall amino acid identity is 41%. The sequence identity between MK2 and MK3 is 75% [2]. MK5 has been found to be ubiquitously expressed throughout the human body, but has a predominant expression in the heart, skeletal muscle, pancreas and lung [4-7]. In resting cells the protein resides predominantly in the nucleus but is able to shuttle between the nucleus and the cytoplasm. Nucleocytoplasmic shuttling is controlled through MK5s interaction with PKA, Cdc15A and the upstream kinases ERK3/4 and p38 [8-16]. The in vivo interaction between p38 and MK5 is, however, under some debate, and is currently not completely resolved (reviewed in [17]).

A lot of experimental work has been performed to elucidate the biological role of MK5. Studies by Tak and coworkers [18], Moens and coworkers [12,16,19], and Choi and co-workers [20] have established the relationship between MK5, hTid-1 and Hsp27 in F-actin rearrangement and cell migration. Several studies have shown the importance of MK5 in cell cycling and proliferation. PRAK was reported to suppress oncogenic RAS-induced proliferation [21], while overexpression of MK5 inhibited proliferation of NIH3T3 cells [22]. MK5 was also found to be essential for ras-induced senescence and thereby to act as a tumor suppressor [23]. Later it was also discovered that MK5 may act as a tumor suppressor through downregulation of Myc [24]. MK5 may also be involved in inhibition of cell proliferation through ERK3 interaction [25,26]. Moreover, MK5 may repress cell invasiveness [27]. Recent studies demonstrated that MK5 can act as a tumor promoter [28]. The authors reported that MK5 stimulates angiogenesis [29]. The same group also unveiled a role of MK5 in cell growth arrest induced by energy starvation [30]. Furthermore, animal studies have suggested that MK5 is involved in neurological processes controlling anxiety and locomotion [31]. Despite all these described functions the exact biological role of MK5 still remains elusive.

A reliable MK5 working model is of great importance in the development of chemical probes to help elucidate the role of MK5. In the present study, we have constructed and refined three MK5 homology models based on three different templates; MK2 ([PDB: 2OZA] and [PDB: 3M2W]) and MK3 [PDB: 3FHR]. The ability of the refined models to effectively discriminate between known inhibitors and decoys was analyzed using receiver operating characteristic (ROC) curves.

## Methods

### Alignment

**Figure 1:**
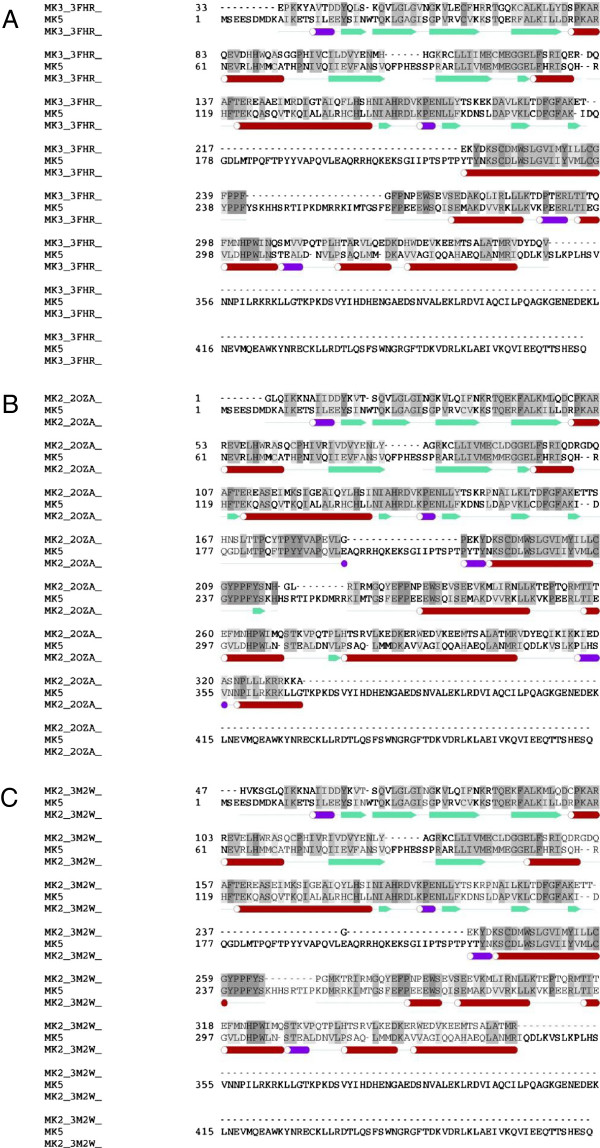
**Alignments. A**: Final alignment used to create homology model of MK5 from MK3 [PDB: 3FHR]. See text for further description on how alignment was obtained. **B**: Final alignment used to create homology model of MK5 from MK2 [PDB: 2OZA]. **C**: Final alignment used to create homology model of MK5 from MK2 [PDB: 3M2W].

Sequence alignments of MK5/MK2 ([PDB: 2OZA] and [PDB: 3M2W]) and MK5/MK3 ([PDB: 3FHR]), respectively, were constructed using ICM’s inbuilt alignment tool. These alignments were adjusted manually to match multiple sequence alignment of the same kinases. The multiple sequence alignment of human [Swiss-Prot: *Q8IW41*], mouse [*O54992*], pig [*B7U6F1*], frog [*Q6DEV6*] and zebrafish [*Q6DHN7*] MK5, and human [*P49137*], mouse [*P49138*], rat [*Q80ZF4*], hamster [*P49136*] and frog [*Q28FG8*] MK2, or human [*Q16644*], mouse [*Q3UMW7*], pig [*B8XSJ7*], rat [*Q66H84*] and bovine [*Q3SYZ2*] MK3 was obtained using T-coffee, Version 4.71 available at the Le Centre National de la Recherche Scientifique website (http://www.igs.cnrs-mrs.fr/Tcoffee/tcoffee_cgi/index.cgi) [32,33]. Finally adjusted alignments (Figures 1A, B and C) were used further for homology modeling.

### Homology modeling

The amino acid sequence of human MK5 (Swiss-Prot: Q8IW41) was retrieved from the Protein Knowledgebase of UniProt (http://www.uniprot.org) [34]. Close homologues with available crystal structures in the Protein Databank (http://www.pdb.org) [35] were identified using BLAST [36] through UniProt. The crystal structure of MK3 in complex with pharmaceutical lead compound P40 [PDB: 3FHR], with a resolution of 1.90Å, and the crystal structure of MK2 complexed with a spiroazetidine-tetracyclic ATP site inhibitor [PDB: 3M2W] with a resolution of 2.4Å, had the highest resolutions of the available MK2 and MK3 crystal structures and were identified as the most suitable template candidates for homology modeling. In addition, the crystal structure of MK2 in complex with p38 [PDB: 2OZA], with a 2.7Å resolution, which was used previously as a template for the construction of an MK5 model [37], was also used as template to construct an MK5 model for comparison. Thus, three MK5 models were constructed based on three different templates; MK2 ([PDB: 2OZA] and [PDB: 3M2W]) and MK3 [PDB: 3FHR].

The homology macro of ICM [38] constructs the backbone of the target molecule by homology from core sections of the template molecule. Core sections are defined by the average of Cα atom positions in these regions, and side chain torsion angles are then predicted by simultaneous global optimization of the energy for all non-identical residues. Loops are subsequently searched for among several thousand structures in the PDB data bank [35] and matched in regard to sequence similarity and steric interaction with the surroundings of the model. Best fitting loops were selected by calculating maps around loops and scoring their relative energies. The C-terminal part, which seems to be unique for MK5, was not included in the models since the corresponding segment was not present in either of the templates. Besides, the main focus of this study was the ATP binding site, and the C-terminal part is not adjacent to the binding site.

The MK5 models were refined using the RefineModel macro of ICM which globally optimizes side chains and anneals the backbone. The macro includes: (1) Monte Carlo fast [39] simulation for sampling of the conformational space of side chains, (2) iterative annealing of the backbone with tethers, which are harmonic restraints pulling an atom in the model to a static point in space represented by a corresponding atom in the template, and (3) a second Monte Carlo fast simulation on side chains. The Monte Carlo method is a complete search of the conformational space of a protein or part of a protein, and at each stage the actual conformation is modified randomly in order to obtain a new one. Each iteration samples the conformational space of a molecule with the ICM global optimization procedure, and consists of a random move followed by a local energy minimization, and then a complete energy calculation. Based on the energy and the temperature, the iteration is accepted or rejected [39].

The electrostatic potential surfaces (EPS) of the 3FHR-based MK5 model, the 3FHR MK3 crystal structure and the 3M2W MK2 crystal structure were calculated with the ICM program, with a potential scale from -5 to +5 kcal/mol using the ICM-REBEL module of the ICM program. This module solves the Poisson equation for a molecule using a boundary element algorithm and generates a 3D surface skin model colored by potential.

### Quality assessment of model/model validation

The SAVS Metaserver for analyzing and validating protein structures (http://nihserver.mbi.ucla.edu/SAVS/) was used to check the stereo chemical quality of the MK5 models. Programs run were ProCheck [40], What_Check [41], and Errat [42]. ProCheck checks stereo chemical quality of the protein structure by analyzing residue-by-residue geometry and overall structure geometry, while What_Check performs extensive checking of many stereo chemical parameters of the residues in the model based on a subset of protein verification tools from the WHAT IF program [43]. Errat analyzes the statistics of non-bonded interactions between different atom types and plots the value of the error function versus position of a 9-residue sliding window, calculated by a comparison with statistics from highly refined structures.

Also the overall 3D structure of the models were checked against their templates, and the RMSD values were calculated utilizing DaliLite (http://www.ebi.ac.uk/Tools/structure/dalilite) [44].

### Inhibitors and decoys

ChEMBL (http://www.ebi.ac.uk/chembl/) was searched for putative MK5 inhibitors, and 316 different compounds with varying ability to inhibit MK5 were identified. Ten of the 316 inhibitors were selected based on known IC50 value (see Table 1) and used as templates to identify decoy substances.

**Table 1:** Putative MK5 inhibitors selected on basis of their IC50 value

**Structure**	**Chemical comp.**	**ChEMBL comp id.**	**Pubmed id.**	**IC50 (nM)**
	C21H18N4OS	CHEMBL1231206	20237073	5
	C24H23N5O2	CHEMBL461139	18945615	24
	C23H23N5O2	CHEMBL461140	18945615	28
	C21H16N4O	CHEMBL226403	17480064	81
	C18H15N3O	CHEMBL388566	17480064	140
	C18H14FN3O	CHEMBL226471	17480064	210
	C12H11N3O	CHEMBL225519	17480064	500
	C30H28O8	CHEMBL34241	10998351	1900
	C13H14N2O3	CHEMBL395157	17570666	19000
	C18H16N6S2	CHEMBL34704	10998351	50000

Decoys were retrieved using the Molcart chemical management system of ICM. Molcart features a number of compound databases (including Ambinter, Chembrigde, Lifechemicals, etc.) that can be analyzed and searched using ICM cheminformatic tools. The decoys were selected based on structural similarities with the known inhibitors. Similarities in molecular mass and in the number of nitrogen- and oxygen atoms were also used as criteria. 924 different decoys were obtained from Molcart and clustered. Ninety decoy substances evenly distributed between the different clusters were chosen and used for docking studies in addition to the ten potential inhibitors (for full list of decoys and inhibitors see Additional file 1). Decoys were chosen from different clusters in order to get as diverse sample as possible.

### Identification of ligand binding pocket

The icmPocketFinder macro of ICM (default tolerance level at 4.6) was used to identify possible binding pockets in the models. The icmPocketFinder algorithm uses a transformation of the Lennard-Jones potential calculated from a three-dimensional protein structure and identifies envelopes according to their shape and physicochemical properties [45]. The algorithm does not require any knowledge about the ligand, and the pockets are based solely on the protein structure. The pocket selected for the docking studies, localized in the ATP binding site of the templates, was identified by icmPocketFinder.

### Docking

In the present study, semi-flexible docking, which keeps the protein itself rigid while the ligand is flexible, was performed. ICM uses a Monte Carlo global optimization procedure [39] to predict binding poses for the ligand in the binding pocket. 2D structures of inhibitors and decoys structures were converted to 3D structures by ICM and automatically assigned charges where necessary. The compounds were then docked into the predicted ATP binding pocket of the different models using the batch docking method of ICM. Three parallel docking runs were performed with each docking procedure and the best score values of the three were kept for evaluation (See Additional files 2, 3, 4).

### Evaluation of docking

The overall predictability of the models was evaluated by ROC curves [46,47]. Inhibitors are labeled 1 (as true inhibitors) and decoys are labeled 0 (as false inhibitors). Score values are then analyzed using the ROC curve script incorporated into ICM. Results are displayed as ROC curves, and the area under curve (AUC) is calculated. A straight line ROC curve would signify that the model gives no preference to true inhibitors over decoys or vice versa. A curve bowing more to the left indicates a greater accuracy of the model (a higher ratio of true positives to false positives).

### Molecular dynamics

In order to refine protein-ligand interactions and to test the stability of the docking pose of the best scored complex from the docking study, a molecular dynamics (MD) simulation was run on the 3FHR-based MK5 model in complex with Ligand 92. The binary complex was first optimized with protein preparation wizard in Maestro V9.1 [48] by assigning bond orders, adding hydrogen and correcting wrong bond types. The system was neutralized by adding 8 Cl^-^ ions. The simulated system consisted of a total of 77439 atoms. A default quick relaxation protocol was used to minimize the MD systems with the Desmond program. In Desmond, the volume of space in which the simulation take place (the global cell) is built up by regular 3D simulation boxes of 10Å X 10Å X 10Å. Each box is assigned to a single Desmond process. The global cell including solute and solvent was of approximately 90Å X 90Å X 90Å. In short, two rounds of steepest descent minimization were performed with a maximum of 2000 steps with and without restrains (force constant of 50 kcal/mol/Å on all solute atoms). This was followed by a series of four MD simulations. The first simulation was run for 12 ps at a temperature of 10 K in the Berendsen NVT (constant number of particles, volume, and temperature) ensemble with solute heavy atoms restrained (force constant of 50 kcal ⁄mol/Å). Next, another 12 ps simulation was performed at 10 K with the same restraints, this time in the Berendsen NPT (constant number of particles, pressure, and temperature) ensemble. With the same restraint, a 12 ps simulation followed with the temperature raised to 300 K in the Berendsen NPT ensemble and the force constant retained. Finally, a 24 ps simulation was performed at 300 K in the Berendsen NPT ensemble with all restraints removed.

After these minimization steps, a total of 50 ns’ MD simulation was performed, using the OPLS 2005 force field [49] at a constant pressure of 1 bar and a temperature of 300 K, using the Nose-Hoover chain [50] and Martyna-Tobias-Klein methods [51]. The short-range and long-range Coulombic interactions were calculated with a cut off radius of 9 Å and with the Smooth particle mesh [52] method (Ewald tolerance: 1e-9). Time step scheduling was used as follows: the multistep RESPA integrator [53] with an inner time step of 2.0 fs for bonded forces and non-bonded near forces within the short range cutoff, an outer time step of 6.0 fs for non-bonded far forces beyond the cutoff.

Atomic distances important for ligand binding were calculated for the entire 50 ns MD simulation. An average model was calculated from frames between 28.7 – 50 ns. This average structure was first optimized by the protein preparation wizard in Schrødinger to correct wrong bond types and overlapping atoms, and then minimized using the Desmond Minimization program. The average model was solvated by a 10Å×10Å×10Å Orthorhombic SPC water box. Maximum number of iterations was 2000 and convergence threshold was 1.0 kcal/mol/Å. Steepest decent minimization was performed until a gradient threshold of 25 kcal/mol/Å was reached, and followed by a limited memory variation of the Broyden–Fletcher–Goldfarb–Shanno (LBFGS) method with vectors 3. The short-range and long-range Coulombic interactions were the same as for the MD simulation. No restraint was applied to the system.

## Results and Discussion

**Figure 2:**
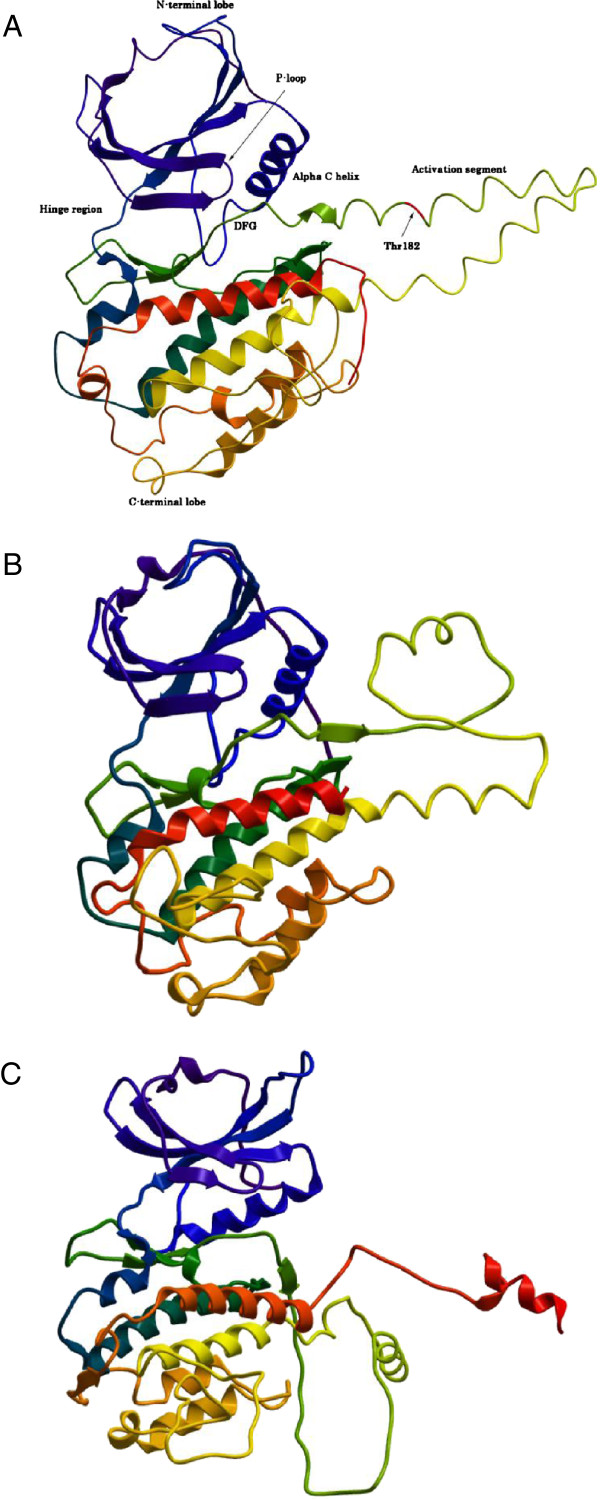
**MK5 models. A**: Final backbone Cα-traces of the MK5 homolog model based on the crystal structure of MK3 (3FHR). The model predicts an N-terminal lobe consisting of a bundle of β-sheets and a conserved α-helix. The C-terminal lobe is largely helical and contains a segment, the activation segment, which includes Thr182 that becomes phosphorylated when MK5 is activated. The hinge region connects the two lobes, and this is where the ATP binding site is predicted to reside. Color coding: Purple via blue, green and yellow to red from N-terminal to C-terminal. **B**: Final backbone Cα-traces of the MK5 homolog model based on the crystal structure of MK2 (3M2W). Color coding as Figure 2A. **C**: Final backbone Cα-traces of the MK5 homolog model based on the crystal structure of MK2 (2OZA). Color coding as Figure 2A.

**Figure 3:**
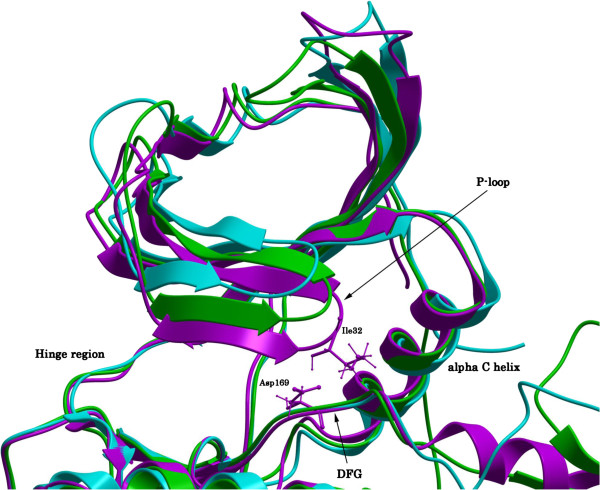
**ATP binding site of superimposed models.** Color coding: 3FHR-based MK5 model: pink; 3M2W-based MK5 model: green; 2OZA-based MK5 model: blue. Important structural parts are marked out with text for easier orientation within the model.

In the absence of an available X-ray crystal structure of MK5, we constructed MK5 homology models based on the structures of its close homologues MK2 (43% sequence identity) and MK3 (41% sequence identity). Three different templates were used; [PDB: 2OZA [54]], [PDB: 3M2W [55]] and [PDB: 3FHR [56]]. The final models are presented in Figures 2A-C. All predicted models have an N-terminal lobe consisting of a bundle of β-sheets, a conserved α-helix and a conserved P-loop. The C-terminal lobe is largely helical and contains the activation segment, including Thr182, which becomes phosphorylated when MK5 is activated. The hinge region connects the two lobes which locate the presumable ATP binding site. The most striking differences between the models are seen in the activation segment of the C-terminal lobe and in the shape of the proposed ATP binding pocket. The 2OZA-based model seems to have a somewhat narrower ATP binding pocket than the other two models, which may be due to the fact that the crystal structure 2OZA is in the apo- (unbound) form, whilst the other two templates have a ligand bound in the ATP pocket. Figure 3 shows the ATP binding sites of the superimposed models. The position of the P-loop was different in the three models. In the 2OZA-based model the gap between Ile32 in the middle of the P-loop and the Asp169 of the DFG motif was 8.68Å. The same distance in the 3M2W- and 3FHR-based MK5 models respectively were of 8.55Å and 6.89Å.

The Ramachandran plot retrieved through ProCheck showed that all models possessed more than 90% of the amino acids in core regions, 9% in allowed regions, and approximately 1% in generously allowed regions. None of the amino acids were in disallowed regions. All models had an Errat option above 85, and together with the ProCheck results, this indicates that the models were of decent stereochemical quality. WhatCheck confirmed that the models are of satisfactory quality. Interestingly, after removal of the flexible loop of the C-terminal lobe, the results given by the SAVS Metaserver tests drastically improved for both the 3FHR- and 3M2W-based models, giving Errat values >90. This indicates that these models were of high quality except for the large flexible loop in the activation segment.

In order to identify structural differences between the models and their templates, the different 3D models were superimposed onto their corresponding template and the RMSD between backbone Cα- atoms were calculated. This was done by the use of DaliLite. The 2OZA-based model has a 46% sequence identity with the template structure 2OZA and an RMSD value of 0.9Å, while the 3M2W-based model has a sequence identity of 46% and an RMSD of 0.8Å. The 3FHR-based model displays a reduced sequence identity of 44% with its template and a slightly lower RMSD value of 0.7Å. Most of the differences between the models and their templates are seen in the flexible loop structures of the activation segment in the C-terminal lobe. Both results obtained from the SAVS Metaserver and DaliLite indicate that the models based on 3FHR and 3M2W are the stereochemically best models.

**Figure 4:**
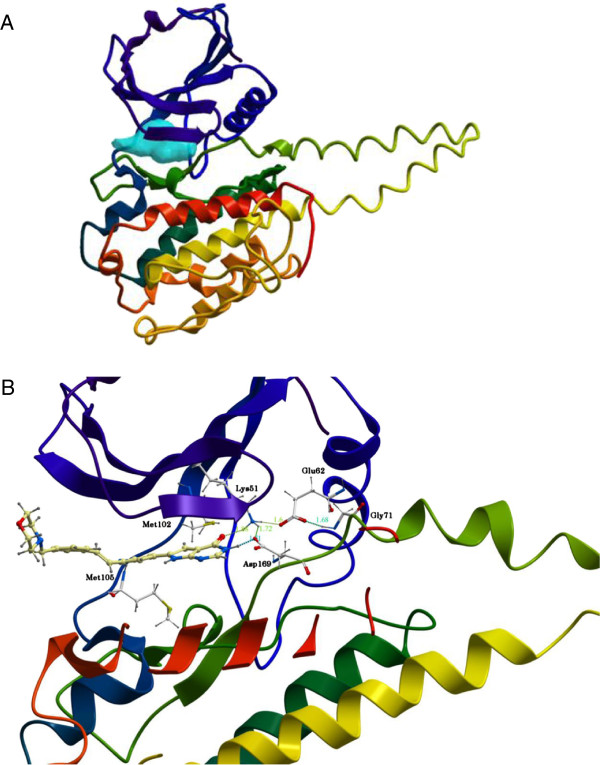
**ATP binding site. A**: Proposed ATP binding site of the 3FHR-based MK5 model. **B**: Ligand 92 docked in proposed ATP binding site of the MK5 model after 50 ns of MD. Color coding: Backbone Cα-traces: Purple via blue, green and yellow to red from N-terminal to C-terminal; Amino acids important for binding of ligand colored according to atom type (C = grey; H = dark grey; O = red; N = blue); Ligand 92: colored according to atom type (C = yellow; H = dark grey; O = red; N = blue). Hydrogen bonds are displayed as dotted lines.

In order to evaluate the models’ ability to distinguish between real inhibitors and decoy substances, the three different models were docked both with the 90 decoy ligands and 10 inhibitors in a semi-flexible mode. Score values from the different dockings ranged from +6 to -36, depending on the model used and the ligand (see Additional files 2, 3, 4 for full list). Figure 4A shows the proposed ATP binding site of the 3FHR-based MK5 model according to ICM Pocket Finder. Common for all of the models was that strong inhibitors had a score value better than -31 and important amino acids for binding of ligands were Lys51, Met105 and Asp169 in the predicted ATP binding site. Ligand 92 had a score value of -36. This compound is predicted to bind closely to the previously mentioned amino acids via hydrogen bonds. Ligand 92 was positioned in the ATP binding cleft between the N- and C-terminal lobes of MK5. It is planar and boomerang shaped with a flexible end, mimicking ATP binding with the pyrimidine ring occupying the same position as seen for the adenine moiety of ATP in MK2 [PDB: 1NY3]. A partial stable hydrogen bond to the backbone amide of Met 105 in the hinge region via the nitrogen of the pyrimidine ring was formed. In addition, the ligand formed a limited number of hydrogen bonds to the active site residues of MK5. The oxygen of the pyrrole-pyrimidin ring interacted with the ϵ-amino group of Lys 51 (1.88Å), and the nitrogen (N5) of the the pyrrole-pyrimidin ring interacted with the carboxyl group of Asp 169 (1.51Å). The active site residues themselves also formed a number of hydrogen bonds contributing to keeping the complex stable. Lys51 formed a hydrogen bond with the carboxyl group of both Asp 169 (1.72Å) and Glu62 (1.6Å), while Glu62 formed an additional hydrogen bond with the nitrogen of Gly71 (1.68Å). The gatekeeper residue Met102 of MK5 did not seem to make contact with Ligand 92. Ligand 92 in the binding site of the 3FHR model after 50 ns of MD is shown in Figure 4B.

The Lysine residue 51 is positioned at the back wall of the ATP-binding niche and is highly conserved amongst mammalian protein kinases. It is believed to be involved in the orientation of the α- and β-phosphate groups of ATP [57]. Mutation of Lys51 to Methionine or Glutamic acid in the ATP pocket of MK5 completely removed the kinase activity [8,9]. Consistent with this we observed that many of the known inhibitors bound to Lys51 in the model or blocked the access of ATP to Lys51.

Comparing the 3FHR- and 3M2W-based models with the active ADP bound crystal structure of MK2 [PDB: 1NY3], revealed that the models appeared to have adopted an active kinase conformation [58], with the catalytic residues Lys51 and Glu62 close enough to make ion pair interaction, and Asp169 in DFG-in conformation, the same conformation as that seen in the active MK2/ADP structure [PDB: 1NY3]. In that conformation the catalytic residues can interact with ATP for phosphate transfer. ADP was docked into the ATP binding sites of the 3FHR- and 3M2W-based models, and it appears that since the 3FHR- and 3M2W-based models originated from complexes with inhibitors, the ATP-binding site was too narrow to accommodate ADP. This was confirmed by comparing the 3FHR- and 3M2W-based models with MK2 [PDB: 1NY3] complexed with ADP; the ATP binding site of the MK2 [PDB: 1NY3] complexed with ADP was slightly wider. The distance between the carboxyl group of Asp169 and Gly31 in the glycine-rich loop in the 3FHR-based model was 5.3Å. In contrast, distance between the carboxyl group of Asp207 and Gly73 in the glycine-rich loop in the MK2 crystal structure [PDB: 1NY3] complexed with ADP was 8.63Å. ADP yielded the same orientation as ADP in the MK2 [PDB: 1NY3] complex, but it was localized approximately 1Å further out from the binding site. The narrowing of the ATP binding site induced by a ligand has also been shown in the crystal structure of MK3 [PDB: 3FHR] complexed with the high-affinity pharmaceutical lead compound (2-(2-quinolin-3-ylpyridin-4-yl)-1,5,6,7-tetrahydro-4H-pyrrolo[3,2-c]pyridin-4-one) (P4O) [56]. In this crystal structure complex, the flexible glycine-rich loop in the binding site occupies the position of the β-phosphate of ADP, thus binding of P4O induces a conformation of a deep and narrow ATP binding pocket in MK3.

**Figure 5:**
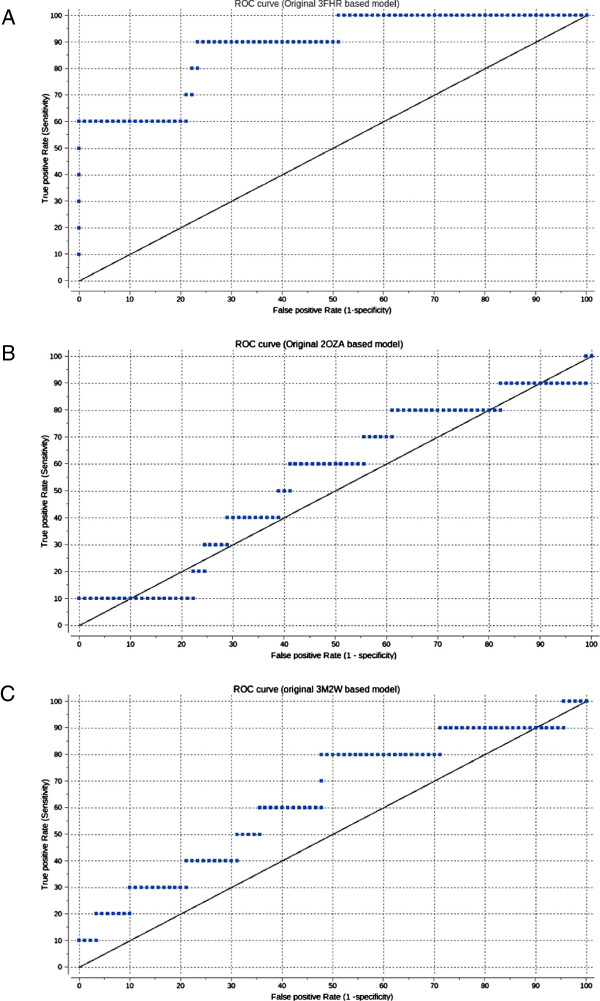
**ROC curves created from the score values. A**: 3FHR-based model. **B**: 2OZA-based model. **C**: 3M2W-based model*.*

The docking results were evaluated both by score values and ROC curves, which describes the tradeoff between “specificity” and “sensitivity”. “Specificity” is the ability to avoid false positives, and “sensitivity” is the ability of the classifier to detect true positives. The area under an ROC curve indicates the quality of enrichment, and while the ROC value of a random classifier is 0.5, the ROC value of an excellent classifier is greater than 0.9 [59]. The 3FHR-based model gives a near ideal ROC curve, with an area under the curve calculated to be 0.88, indicating this model performs very well for predicting ligand binding to the ATP site (Figure 5A). The 2OZA- and 3M2W-based models (Figure 5B and C), however, give a graph close to the diagonal line (with an area under curve calculated to 0.67), indicating that these models gave a somewhat random result and are less suited for predicting ligand binding than the 3FHR-based model.

Both 3FHR and 2M2W are crystal structures of kinases with a ligand in the ATP binding site. 2OZA on the other hand is in apo- (unbound) form bound to the upstream kinase p38. The models built reflect this difference in template by having a much narrower ATP binding pocket for the 2OZA-based model than the others. 2OZA is in apo- (unbound) form, which probably explains why 2OZA is an unsuitable template for constructing an active MK5 model that can be used to identify chemical probes that bind the ATP pocket. This was also obvious when docking known inhibitors into this model. The pocket is too narrow, and poor scoring values were obtained. The ROC curves also indicate that the model was not able to distinguish between known inhibitors and decoys. However, the 2OZA model may be suitable for identification of possible type II kinase inhibitors that typically bind to kinases in the inactive state [60].

**Figure 6:**
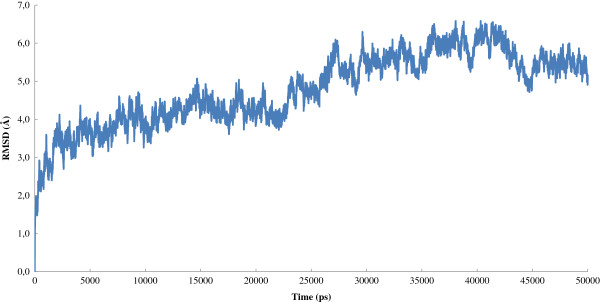
Graph of the RMSD of the backbone of the 3FHR-based MK5 model in complex with Ligand 92 during the 50 ns of MD simulation.

**Figure 7:**
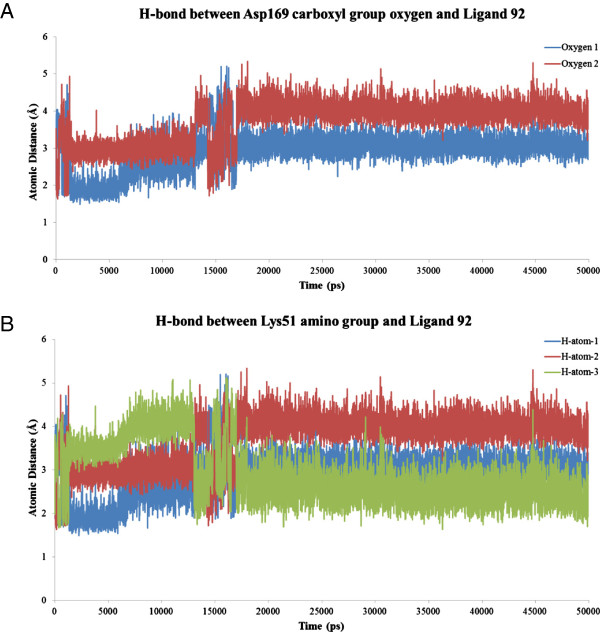
**Plots of atomic distance variations. A**: Plot of the atomic distance variation of the hydrogen bonds between the Asp169 carboxyl group oxygen atoms and Ligand 92 during the 50 ns of MD simulation. **B**: Plot of the atomic distance variation of the hydrogen bonds between the Lys51 amino group and Ligand 92.

Figure 6 shows the graph of the RMSD of the backbone of the 3FHR-based MK5 model in complex with Ligand 92 during the 50 ns of MD simulation. The average 3FHR-based MK5 model during the last 21.3 ns of MD simulation was superimposed onto the initial 3FHR-based MK5 model, and the RMSD value was 0.803, indicating a very stable MK5-ligand complex. Figure 7A shows a plot of the atomic distance variation of the hydrogen bonds between the Asp169 carboxyl group oxygen atoms and Ligand 92 during the 50 ns of MD simulation, and Figure 7B shows a plot of the atomic distance variation of the hydrogen bonds between the Lys51 amino group and Ligand 92. The plot indicates that hydrogen atom 1 and 3 exchanges places as participants in the hydrogen bond between the Lys51 amino group and Ligand 92 during the simulation. The Ramachandran plot of the average structure is shown as an additional figure (Additional file 5). A video of the 50 ns of MD simulation of the 3FHR-based MK5 model in complex with Ligand 92 (Additional file 6) indicates that the complex is generally stable. Observable dynamic flexibility of the binary complex (from video) also features that from 22 ns to 50 ns, the terminal sequence Met340 – Ser348 is turning inward to the active site direction, and from 20 ns to 50 ns the loop between Gln87 and Pro94 is turning downwards towards the active site. From 22 ns to 50 ns, the helix-loop-helix stretch between Ala191 and Lys205 appears to be flexible, and from 0 ns to 50 ns, the longest loop, between Gly237 and Ser274 is very flexible.

**Figure 8:**
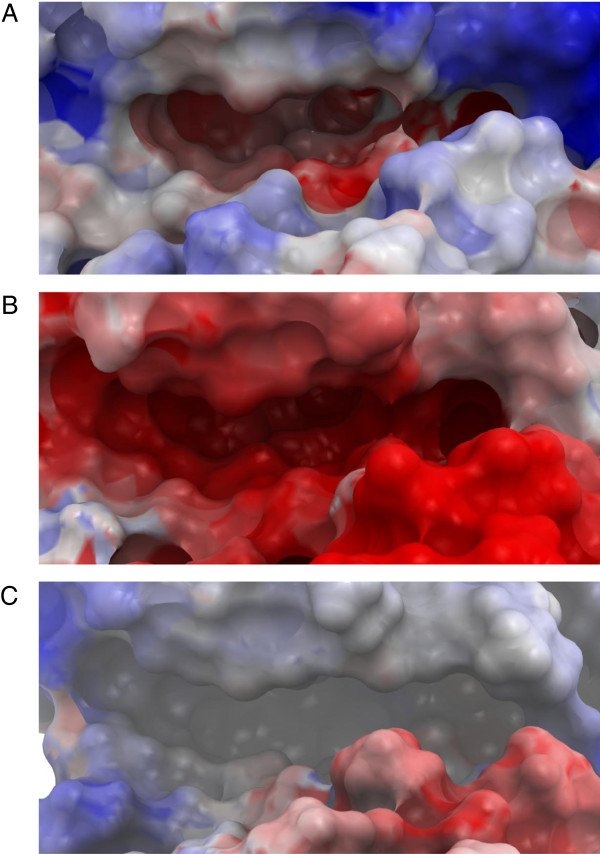
**EPS of ATP binding sites. A**: EPS of the ATP binding site of the 3FHR-based MK5 model. **B**: EPS of the ATP binding site of the 3FHR MK3 crystal structure. **C**: EPS of the ATP binding site of the 3M2W MK2 crystal structure. Color coding: negative (-5 kcal/mol) red, via white, to positive (+5 kcal/mol): blue.

**Figure 9:**
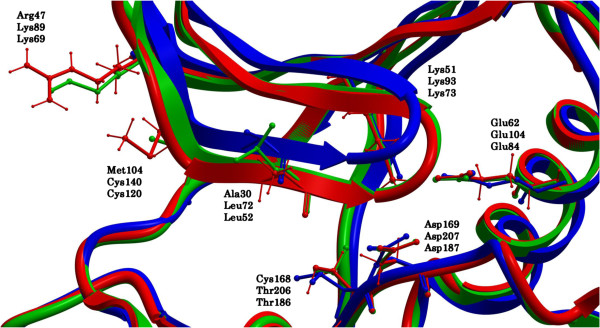
**Comparison of ATP binding sites of MK5 model, and MK2 (3M2W) and MK3 (3FHR) crystal structures.** Important amino acids for ligand interaction are labeled at tip of residues. Constructed model based on 3FHR is colored red, 3M2W crystal structure is colored blue and 3FHR crystal structure is colored green.

The main challenge in developing new chemical probes towards protein kinases is selectivity of the probes in order to discriminate between the different protein kinases. At the moment no selective MK5 inhibitors are known, and the selected inhibitors in the present docking study are also able to inhibit MK2 and MK3. This, however, is not important at the present stage since the model is only meant to be able to identify binders versus non binders. The present 3FHR-based MK5 model can be used to elucidate the uniqueness of the MK5 ATP-binding site and may serve as a working tool for developing chemical probes using interactive drug design. Figures 8A, B and C show the EPS of the ATP binding sites of the 3FHR-based MK5 model, the 3FHR MK3 crystal structure and the 3M2W MK2 crystal structure. The EPS of the ATP binding site of the 3FHR-based MK5 model appears to be electronegative in its two deeper cavities and electropositive in its outer areas. The EPS of the ATP binding site of the 3FHR MK3 crystal structure is entirely electronegative, and the EPS of the ATP binding site of the 3M2W MK2 crystal structure is partly electropositive and partly electronegative. The sizes of the cavities of the 3FHR-based MK5 model, the 3FHR MK3 crystal structure and the 3M2W MK2 crystal structure, measured using ICM Pocket Finder, were 420Å^3^, 384Å^3^ and 331Å^3^, respectively. Amino acids Asp169, Lys51 and Glu62 (numbers related to MK5), which are important for the binding and phosphate transfer from ATP [58], are located in the similar positions in all three kinases. There are, however, some differences, the most important being Cys168 in MK5 (Figure 9). In the corresponding position, MK2 and MK3 both have a threonine. A cysteine in the ATP binding site may represent a unique opportunity for the construction of irreversible covalent inhibitors to the nucleophilic cysteine residue [61]. Development of irreversible inhibitors that form covalent bonds with cysteine in the ATP-binding pocket is currently gaining interest [62]. Irreversible kinase inhibitors may have potential advantages including prolonged pharmacodynamics and suitability for rational drug design. Other amino acids in the ATP binding site of MK5 that are different from the corresponding positions in both MK2 and MK3 are Ala30 (leucine in MK2 and MK3), Arg47 (lysine in MK2 and MK3), and Met104 (cysteine in MK2 and MK3) (Table 2).

**Table 2:** Result from comparison of ATP binding site of MK2, MK3 and MK5

**MK5 model**	**MK2_2OZA (fastafile)**	**MK2_3M2W (fastafile)**	**MK3_3FHR (fastafile)**
Q26	Q68	Q68	Q48
L28	L70	L70	L50
G29	G71	G71	G51
A30	L72	L72	L52
G31	G73	G73	G53
I32	I74	I74	V54
V36	V78	V78	V58
R47	K89	K89	K69
A49	A91	A91	A71
K51* [8,9]	K93	K93	K73
E62*	E104	E104	E84
V76	V118	V118	V98
M102**	M138	M138	M118
E103	E139	E139	E119
M104	C140	C140	C120
M105	L141	L141	M121
E106	D142	D142	E122
G107	G143	G143	G123
G108	G144	G144	G124
E152	E190	E190	E170
N153	N191	N191	N171
L155	L193	L193	L172
C168	T206	T206	T186
D169*	D207	D207	D187

* Amino acid important for binding of ATP [58].

** Gate keeper residue.

## Conclusions

Even though the biological function of MK5 still remains elusive, it will be interesting to see how its role as a possible drug target may be elucidated in the near future. The 3FHR-based model presented in this study was effective in discrimination between known inhibitors and decoys, being capable of discriminating between 10 actives and 90 property-matched decoys at 88% level. This indicates that this model may be used as a working tool for further experimental studies and possibly structure aided drug design. Given the tumor suppressor and tumor promoting properties of active MK5 (reviewed in Kostenko et al. [63]), the design of specific MK5 activators and inhibitors may have important therapeutic potentials.

Coordinates of MK5 models and protein-ligand complexes are available upon request.

## Competing interests

The authors declare that they have no competing interests.

## Authors’ contributions

IL created the sequence alignments, carried out the molecular modeling studies (homology modeling, refinement and validation), tested models against inhibitors and decoys, and drafted the manuscript. YW carried out the MD simulations. IK and RA contributed with molecular modeling advice and critical review of the manuscript. UM helped design the study and brought valuable biological advice as well as a critical review of the manuscript. IS and AWR participated in the design of the study, bioinformatics advice and critical review of the manuscript. All authors have read and approved the final manuscript.

## Supplementary Material

Additional file 1 Inhibitors and decoys. Click here for file

Additional file 2 2OZA-based MK5 model and docking. Click here for file

Additional file 3 3FHR-based MK5 model and docking. Click here for file

Additional file 4 3M2W-based MK5 model and docking. Click here for file

Additional file 5 Ramachandran plot of the average MD structure. Click here for file

Additional file 6 Video of 50 ns of MD simulation of the 3FHR-based MK5 model in complex with Ligand 92. Click here for file
