# Supplementary material for: Homology modeling and ligand docking of Mitogen-activated protein kinase-activated protein kinase 5 (MK5)
Source: Theor Biol Med Model. 2013 Sep 14;10:56. doi: 10.1186/1742-4682-10-56 (PMC3848485; doi:10.1186/1742-4682-10-56)
Supplement: Additional file 5 — Ramachandran plot of the average MD structure. [file 1742-4682-10-56-S5.pdf]

PROCHECK

Ramachandran Plot

Average

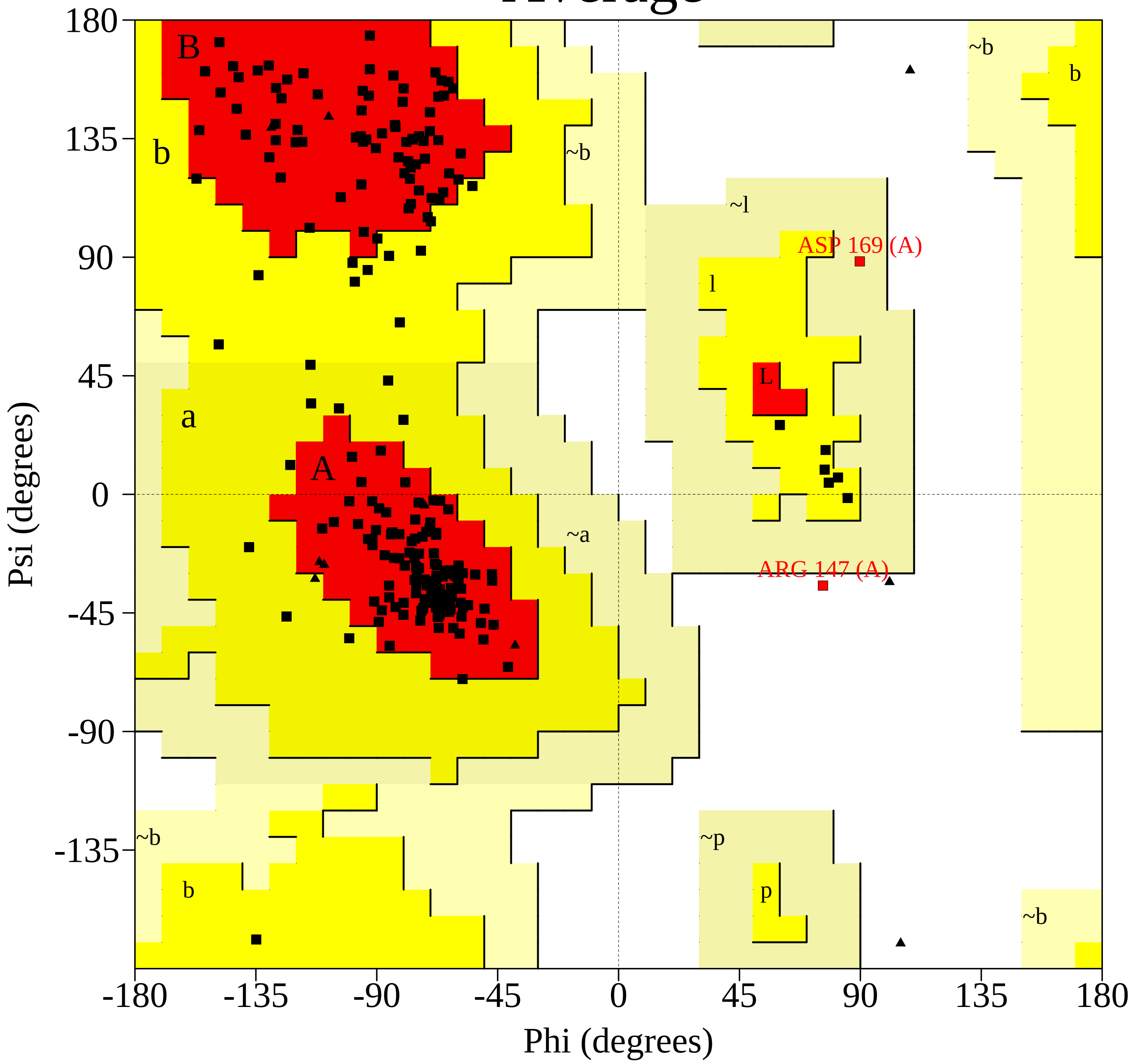

Plot statistics

Residues in most favoured regions [A,B,L]	187	85.4%
Residues in additional allowed regions [a,b,l,p]	30	13.7%
Residues in generously allowed regions [~a,~b,~l,~p]	1	0.5%
Residues in disallowed regions	1	0.5%
	----	------
Number of non-glycine and non-proline residues	219	100.0%
Number of end-residues (excl. Gly and Pro)	89	
Number of glycine residues (shown as triangles)	13	
Number of proline residues	20	

Total number of residues	341	

Based on an analysis of 118 structures of resolution of at least 2.0 Angstroms and R-factor no greater than 20%, a good quality model would be expected to have over 90% in the most favoured regions.

Average_01.ps
